# Editorial: Developmental trajectories of early life trauma

**DOI:** 10.3389/fpsyt.2024.1548431

**Published:** 2025-01-21

**Authors:** Sujata Sethi, Naseem Akhtar Quershi, Giorgio Di Lorenzo

**Affiliations:** ^1^ Department of Psychiatry, Pandit Bhagwat Dayal Sharma University of Health Sciences, Rohtak, India; ^2^ Department of Psychiatry, Al Fatah School of Medical Sciences & Research, Faridabad, India; ^3^ Department of Systems Medicine, University of Rome Tor Vergata, Rome, Italy; ^4^ IRCCS Fondazione Santa Lucia, Rome, Italy

**Keywords:** early, life, trauma, stress, developmental, adulthood

Going through the articles chosen for the Research Topic “Developmental Trajectories of Early Life Trauma”, I was reminded of a quote by Dr. Asa Don Brown, “Childhood trauma doesn’t come in one single package” (1).

Trauma experienced in early life leaves an indelible mark on an individual’s psychological and emotional well-being, often shaping their mental health trajectory well into adulthood and old age. Early life experiences represent an important influence on children’s neural, behavioral, and psychological development, having long-lasting effects across a wide range of domains (2, 3).

An early experience that has garnered much attention is that of chronic and/or extreme stress in early life. Experiences of chronic and/or severe stress during early childhood, often also conceptualized as early life trauma, have persistent and pervasive consequences for development (4, 5).

Childhood trauma victims exhibit low self-esteem, and experience depression and anxiety and engage in alcohol and other drugs in attempts to prevent their traumatic experiences from impacting their life (6).

The articles in this Research Topic delve into the multifaceted impact of early-life adversity, shedding light on how such experiences influence the development and persistence of psychiatric disorders, co-morbidities, and quality of life.

This Research Topic represents a significant step in understanding trauma-induced psychiatric conditions and highlights the need for innovative therapeutic strategies to mitigate these adverse effects. From non-suicidal self-injury (NSSI) among prisoners to the nuanced impact of adverse childhood experiences (ACE) on adulthood behaviors, the breadth of studies here underscores the pervasive nature of trauma and its repercussions.

## Insights from the research

Li et al.’s work on *Childhood maltreatment and NSSI in prisoners* demonstrates the intricate interplay between environmental and intrapersonal factors. The study reveals how self-identity mediates the effects of childhood maltreatment on NSSI and how sensation-seeking can modulate this relationship. This research emphasizes the importance of tailoring interventions to address these interconnected factors in high-risk populations.

Landa-Blanco et al. address the alarming prevalence of ACE in Honduras, exploring their association with depression, anxiety, and maladaptive behaviours such as sexual risk-taking and alcohol consumption. Their findings highlight the need for public health initiatives to reduce ACE and provide targeted mental health support to vulnerable populations.

Sood et al. employ bibliometric analysis to map the landscape of stress and mental health issues among primary and middle school students. By identifying trends such as cyberbullying and test anxiety, the study underscores the pressing need for early interventions to address stress in young learners, particularly in the digital age.

Mohammadi et al. focus on complex PTSD and related symptoms across developmental stages using network analysis. Their study identifies disturbance in self-organization as a central psychopathological symptom, emphasizing the role of adolescent trauma in symptom manifestation. This work highlights the importance of early detection and of trauma-focused therapies in adolescence.

Rizeq et al. test the stress sensitization hypothesis, exploring the impact of early childhood deprivation on vulnerability to later life stressors. Their findings reveal that peer-related life events exacerbate emotional problems among individuals with a history of extreme deprivation. This nuanced understanding calls for targeted interventions addressing peer relationships in adolescence for those with early-life trauma.

## Toward better interventions and understanding

The studies in this Research Topic collectively underline the complexity of trauma-induced psychiatric disorders and their evolution over time. They call attention to the need for longitudinal research, comprehensive diagnostic approaches, and multifaceted treatment modalities—including pharmacological and non-pharmacological interventions—to alleviate symptoms and improve patient outcomes.

Moreover, they point to the significance of addressing psychiatric and non-psychiatric co-morbidities, which often exacerbate the burden of primary disorders. As these articles show, the interplay of psychological, social, and biological factors requires a multidisciplinary approach to care.

## Looking ahead

This Research Topic contributes to a growing body of evidence that early-life trauma has far-reaching implications for mental health. It is imperative to translate these insights into practice by advocating for trauma-informed care models, policy reforms to mitigate childhood adversity, and enhanced accessibility to mental health resources.

As editors, we commend the authors for their rigorous research and meaningful contributions to this vital field. We invite readers to explore these articles and join the discourse on how we can collectively work to address the long shadow of early-life trauma. We hope this Research Topic inspires new research and innovative interventions, fostering resilience and recovery for those affected by trauma.
